# Fatty Acid Binding Protein 1 is an Independent Prognostic Biomarker for Gallbladder Cancer with Direct Hepatic Invasion

**DOI:** 10.7150/ijms.93413

**Published:** 2024-03-25

**Authors:** Shimei Qiu, Zhaonan Liu, Jun Hu, Ziyi Wang, Zhuying Yue, Ziheng Jia, Wenhua Zhang, Ziru Xue, Zebing Liu, Yingbin Liu

**Affiliations:** 1 School of Health Science and Engineering, University of Shanghai for Science and Technology, Shanghai, 200093, China.; 2 Department of Biliary-Pancreatic Surgery, Ren Ji Hospital, Shanghai Jiao Tong University School of Medicine, Shanghai, 200127, China.; 3 Department of Animal Science and Technology, Shanghai Vocational College of Agriculture and Forestry, Shanghai, 201699, China.; 4 State Key Laboratory of Systems Medicine for Cancer, Shanghai Cancer Institute, Renji Hospital, Shanghai Jiao Tong University School of Medicine, Shanghai, 200032, China.; 5 Department of Pathology, Ren Ji Hospital, Shanghai Jiao Tong University School of Medicine, Shanghai, 200127, China.

**Keywords:** FABP1, Gallbladder cancer, Direct Hepatic Invasion, Metastasis, Prognosis

## Abstract

**Background:** Direct liver invasion (DI) is a predominant pathway of gallbladder cancer (GBC) metastasis, but the molecular alterations associated with DI remain addressed. This study identified specific genes correlated with DI, which may offer a potential biomarker for the diagnosis and prognosis of advanced GBC.

**Methods:** RNA samples from 3 patients with DI of GBC were used for RNA-seq analysis. Differentially expressed genes and metabolic pathways between primary tumor (T) and DI tissue was used to analyze aberrant gene expressions. Immunohistochemistry (IHC) of fatty acid binding protein 1 (FABP1) in 62 patients with DI was engaged to evaluate its association with clinicopathological characteristics and prognosis. IHC of CD3^+^ and CD8^+^ T cells was analyzed for their correlation with FABP1 expression, clinicopathological features and prognosis. Univariate and multivariate Cox hazards regression analyses were performed to identify independent prognostic factors for disease-free survival (DFS) and overall survival (OS).

**Results:** FABP1 mRNA levels were significantly upregulated in DI region compared to T tissue. IHC results showed identical results with elevated FABP1 (p < 0.0001). Expression of FABP1 in DI region was significantly associated with lymph node metastasis (P = 0.028), reduced DFS (P = 0.013) and OS (P = 0.022); in contrast, its expression in T region was not associated with clinicopathological characteristics and prognosis (P > 0.05). The density of CD8^+^ T cells in DI region with higher FABP1 expression was significantly lower than that with lower FABP1 expression (p = 0.0084). Multivariate analysis unveiled those hepatic metastatic nodules (HR = 3.35, 95%CI: 1.37-8.15, P = 0.008) and FABP1 expression in DI region (HR = 2.01, 95%CI: 1.05-3.88, P = 0.036) were high risk factors for OS, and FABP1(HR = 2.05, 95%CI: 1.04-4.06, P = 0.039) was also a high risk factor for DFS.

**Conclusions:** Elevated expression of FABP1 in DI region serves as a potential prognostic biomarker for advanced GBC with DI.

## Introduction

Gallbladder cancer (GBC) is the most common malignant tumor of the biliary tract, which is characterized with high invasiveness, rapid metastasis and poor prognosis 1-3. Because there is lack of typical symptoms at the early stage of GBC, the high risk factors associated with carcinogenesis and development of GBC such as long-standing chronic inflammation, gallstones and gallbladder polyps have received significant attention in the diagnosis and treatment 4, 5. Only 25% of patients with GBC meet the qualification to undergo surgical resection, although the radical surgical removal is still at present the most effective method to treat GBC 6, 7. Most patients with GBC are discovered at advanced stages or metastatic stage during which they have missed the surgical opportunity of tumor resection. Liver metastasis is the most common malignant form of GBC metastases, while regional lymph node metastasis also often occurs 8. The hepatic metastases are pathologically classified into 3 types: direct invasion through the gallbladder bed, hepatic metastatic nodules (blood circulation), and portal tract invasion (lymph system) 9. Given the anatomic unique of the gallbladder which is located below the right lobe of the liver, GBCs are directly accessible to the hepatic parenchyma where cancer cells invade, committing to the most common pathway of hepatic invasion. The second common hepatic spread of GBC is attributed to the development of several small veins in the connective tissue between the gallbladder and the liver by which GBCs are able to travel to the liver IV b and V segments 10. Alternatively, tumor cells can also invade the portal tract by lymphatic spread 11. Although the radiotherapy and chemotherapy are the mainstream of the therapeutic intervention except surgery, the efficacy is largely limited due to rapid development of radio/drug resistance. Even for the small population of patients that meet to receive surgical treatment, disease relapse develops frequently, demonstrating the poor prognosis of GBC. Therefore, identification of reliable biomarkers is of great value in clinic for the treatment selection and prognosis prediction of GBC with liver metastasis.

Fatty acid binding proteins (FABPs) comprise a family of 14-15 kDa cytoplasmic lipid chaperones that bind to a variety of fatty acids (FAs) and other intracellular hydrophobic ligand, and transport them into multiple subcellular compartments such as peroxisomes, mitochondria, endoplasmic reticulum and nucleus 12-15. Three members of FABPs are identified in three corresponding organs and/or tissues, in which liver FABP (L-FABP, also known as FABP1) is expressed in the liver, heart FABP (H-FABP, also known as FABP3) in the heart and intestinal FABP (I-FABP, also known as FABP2) in the intestine 16. In cancer cells, FABPs were found to promote the uptake of fatty acids to mediate lipid metabolism of cancer cells during metastasis 17. FABP1 overexpression can induce angiogenesis and migration of hepatocellular cancer cells, leading to increased liver metastases 18. FABP1 has been reported as a potential target for HCC chemotherapy 19. In addition, FABP1 is highly expressed in 44% of melanoma patients and the acquired expression of FABP1 increases uptake of adipocytic derived lipids, rendering melanoma cells highly invasive 20. Interestingly, in gastric cancer patients, FABP1 is also highly expressed in gastric adenocarcinoma tissues, while it is not or less expressed in normal gastric tissues 21. In breast cancer, increased expression of FABP1 induces fatty acid synthase (FASN) that mediates epithelial-mesenchymal transition, thus promoting EMT 22. Although FABP1 has been intensively studied in a number of human cancers, its pathological role in GBC and its predictive potential for disease outcome remain unknown.

In this study, we compared the gene expression profiles between direct liver invasion (DI) and primary tumor (T) of GBC by analyzing the differentially expressed genes (DEGs). FABP1 was elevated in hepatic invasion and its expression levels were negatively correlated with infiltrating CD8^+^ cytotoxic T lymphocytes, indicating the poor prognosis. This study may offer novel potential value for FABP1 in the diagnosis and prognosis of GBC with hepatic metastasis.

## Materials and Methods

### Patients and samples

A total of 62 patients diagnosed with GBC with DI after surgical resection were enrolled in our study in Renji Hospital, in which 13 cases were also found with liver nodal metastases. The study was approved by the human ethics committee of Renji Hospital. All of recruited patients did not receive any antitumor therapy before surgery. Patients' clinical information was collected and catalogued separately including age, sex, tumor size, pathological diagnosis and date of surgery. Samples from three GBC patients with only DI metastasis were cryopreserved in liquid nitrogen for subsequent RNA sequencing. Paraffin-embedded tissues from all 62 patients were sectioned for immunohistochemical (IHC) staining. Survival information for outpatients was usually collected through clinical visits or phone calling communication. Disease-free survival (DFS) from 49 cases was determined from the time of treatment to the recurrence of disease (or death) after undergoing curative-intent treatment. Overall survival (OS) from 60 cases was determined from the time between the surgery and either the date of death or last collected information.

### Hematoxylin-eosin staining and evaluation

Formalin-fixed and paraffin-embedded (FFPE) tissue samples were cut into 3 µm sections. After baked at 65 °C for 1 hour, the sections were deparaffinized with xylene and sequentially immersed into high to low concentrations of ethanol (100%, 95% to 75%). After washing, the sections were stained with hematoxylin for 5 minutes and 1% acid ethanol for three seconds. Sections were rinsed with running water for 5 minutes and subsequently immersed in eosin for 3 minutes. The slides were sequentially immersed in 75%, 95%, 100% ethanol solutions and xylene for dehydration. The slides were then covered with coverslips and sealed with neutral gum prior to observation under a microscope. All hematoxylin and eosin (H&E) stained slides were reviewed independently by two pathologists. All 62 patients' T and DI samples were evaluated and confirmed. For DI samples, we selected liver invaded tissue at least 5 mm width on the border between malignant cells and liver tissue. In addition, these samples were classified according to different histological features that include pathological subtypes, tumor differentiation, perineural invasion, lymphatic invasion, hepatic metastatic nodules and distant metastasis. Tissue samples with extensive necrosis of tumor tissues were excluded.

### IHC staining

IHC staining for FABP1, CD3 and CD8 was performed on sections of all 62 DI tumor samples. FABP1 IHC staining was also processed on all primary tumors. All sections were baked at 65 °C for 1 hour and then processed on an automated IHC Staining System (Leica Bond-Rx; Leica Biosystems). Firstly, the sections were deparaffinized in Dewax Solution and hydrated through ethanol and Wash Solution. Next, antigen retrieval was performed. The sections were boiled in ER2 (pH 9.0; FABP1) or ER1 (pH 6.0; CD3 and CD8) solution for 20 minutes. The sections were incubated in Peroxide Block (Bond Polymer Refine Detection Kit, Leica Biosystems) for 5 minutes to block endogenous peroxidase. Then, the sections were washed three times in wash buffer and incubated with a dilution of primary antibodies for 30 minutes at 37 °C. Detailed information of primary antibodies and conditions used in IHC assays were provided in Supplementary Table S1. After thoroughly washed, the samples were incubated for 10 minutes with different secondary antibodies. The sections were incubated with DAB mixture solution and nuclei were counterstained with hematoxylin. DAB, hematoxylin and secondary antibodies were included in Refine Detection Kit (Leica Biosystems, DS9800). Samples were finally rinsed with water, treated with ethanol, covered, and sealed. The slides were observed under a microscope. Positive and negative controls were included in each IHC assay. Normal tonsil tissues served as positive controls for CD3 and CD8 IHC staining, while colon tissues were utilized as positive controls for FABP1 IHC staining. Additionally, samples incubated with the antibody diluent under the same experimental conditions served as negative controls.

### Evaluation of IHC

The expression of FABP1 was independently evaluated by two researchers who were not allowed to access to patients' medical records. If the evaluated results were inconsistent, a pathologist was requested to assess the samples independently. In these cases, the data from the majority (two out of three) were determined as the final result. FABP1 expression from T and DI was evaluated in all 62 cases. The expression of FABP1 is located in the cytoplasm. Staining quantification was performed according to the following criteria 23: (1) staining intensity as follows: score 0, negative; score 1, weakly positive; score 2, moderately positive and score 3, strongly positive. (2) percentage of staining as follows: score 0, <10%; score 1, 1%-25%; score 2, 25%-50% and score 3, >50%. The total score from 0-9 was calculated by multiplying “staining intensity score” and “staining percentages score”. Thus, the final combined scores determined the staining levels: score 0, negative; score 1-2, weak positive staining; score 3-5, moderate positive staining; score 6-9, strong positive staining.

Tumor-infiltrating lymphocytes (TILs) were assessed by calculating the density of cells (cells/mm^2^) expressing a given marker (CD3 or CD8) in DI subregion. The stained slides were scanned at 200x original magnification by Aperio AT Turbo scanner (Leica Biosystems, USA). Five random regions contained with more than 50% of tumor cells were annotated using Aperio ImageScope software v12.1 (Leica Biosystems, USA), and analyzed for the density of infiltrated cells using the Nuclear v9 algorithm.

### RNA-seq and analysis

Total RNA was extracted using RNeasy Mini Kit following the manufacturer's instructions and analyzed for RNA integrity by an Agilent Bioanalyzer 2100. Qualified total RNA was further purified by RNA Clean XP Kit and RNase-Free DNase Set. Library construction was performed using the VAHTS Universal V6 RNA-seq Library Prep Kit for Illumina. The concentration of the constructed library was detected using Qubit® 2.0 Fluorometer, and the size of the library was detected using Agilent4200. Two-end sequencing was performed on an Illumina sequencer.

For RNA-seq analysis, we used Trim Galore!to detect and trim adapters automatically. The sequencing reads were then mapped to the hg38 genome using Hisat2 24. Read counts were obtained with HTSeq (v0.11.1) 25. We identified differentially expressed genes using DESeq2 (v1.34.0) 26. Expression with | Log fold change | > 2 and adjusted p value < 0.05 were considered to be significant differential expression.

### Statistical analysis

Statistical analyses were performed using GraphPad Prism 8 and R software. Categorical variables were compared using the chi-square test or Fisher's exact test. Continuous variables were compared using the t test or Mann-Whitney U test. Survival curves were plotted using the Kaplan-Meier method and compared using the log-rank test. The Cox proportional hazard regression model was used for the univariate and multivariate analysis. All tests were 2-sided and p values <0.05 were considered statistically significant levels.

## Results

### Clinicopathological characteristics of patients

The study cohorts consisted of 62 GBC patients with DI. Patients' demographics and clinicopathologic characteristics were shown in Supplementary Table S2. The median age at diagnosis of GBC patients was 67 years (IQR, 60-71). The male/female ratio was 1:1.4. Almost half patients had a history of gallstone. Clinical staging was evaluated according to the eighth edition of the TNM staging system defined by the AJCC. All Patients were classified into T3 (n=50, 80.65%) and T4 (n=12, 19.35%) stages, and 65.45% of them developed lymph node metastasis. The TNM stage was III (n = 28, 45.16%) or IV (n = 34, 54.84%). The primary tumor subtype of the patients was adenocarcinoma (n=49, 79.03%) and other tumor subtypes included neuroendocrine carcinoma, mixed adenoneuroendocrine carcinoma, adenosquamous carcinoma and undifferentiated carcinoma. Perineural invasion (n=28, 45.16%) was frequently observed in these cases.

### Transcriptome analysis of liver invasion

**Figure 1:**
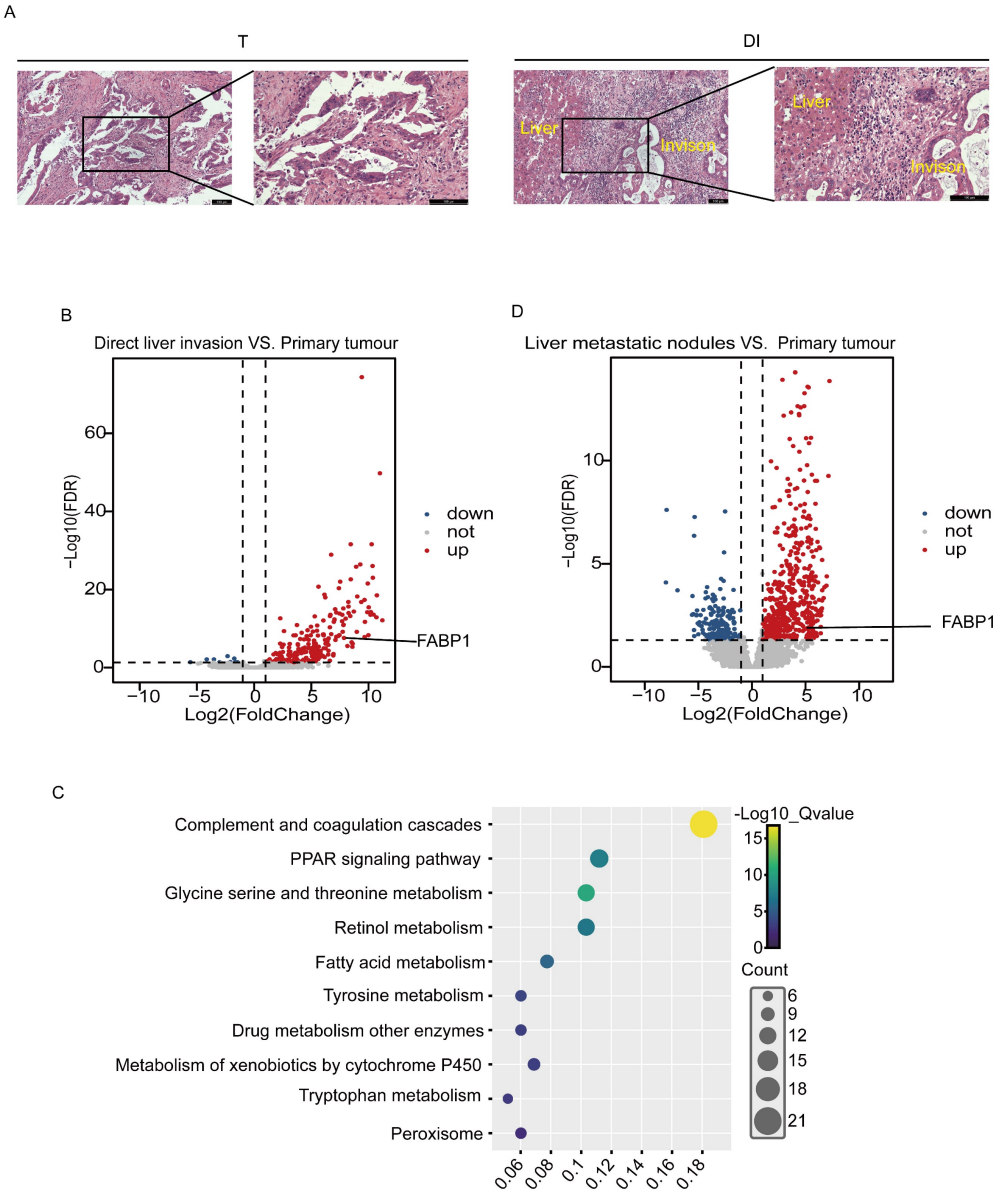
Comparison of transcript/mRNA levels in GBC tissues with primary tumors and those with liver invasion. **(A)** Representative HE images of different regions of patients with GBC (100X and 200X). Bar: 100 um. **(B)** Volcano plots of differential gene expression between direct liver invasion (DI) and paired primary tumor (T) from 3 GBC patients. **(C)** Kyoto Encyclopedia of Genes and Genomes (KEGG) enrichment analysis of upregulated pathways in DI vs. paired T. **(D)** Volcano plots of differential gene expression between paired liver metastatic nodules and primary tumor samples from public dataset with liver metastasis patients (GSE132223). Each circle represents a gene. Red color refers to significantly upregulated genes (Log fold change > 2 and adjusted p value < 0.05) while blue color refers to significantly downregulated genes (Log fold change < -2 and adjusted p value < 0.05).

The frozen tissues from 3 cases of GBC with DI were collected and examined by histopathological morphology in the laboratory, and tumor tissues were identified in the T and DI (Figure 1A). RNA sequencing was then processed in the T and DI regions to identify specific oncogenes potentially associated with liver invasion. We identified 255 DEGs from paired DI and T region, in which 245 genes were up-regulated and 10 genes were down-regulated (Figure 1B). Pathway enrichment analysis through Kyoto Encyclopedia of Genes and Genomes (KEGG) revealed that these DEGs were involved in immune and metabolic pathways including complement and coagulation cascade, PPAR signaling pathway and fatty acid metabolism (Figure 1C). PPAR signaling has been widely reported to be associated with cancer metastasis and metabolism 27-29. In the up-regulated DEGs enriched in the PPAR signaling pathway, fatty acid binding protein 1 (FABP1) that was appreciated to mediate cancer lipid metabolism had raised our particular attention. FABP1 has been reported to increase tumor metastasis by promoting angiogenesis in hepatocellular carcinoma 18. To further confirm this gene involved in liver metastasis, we screened DEGs in the public GEO database with 3 GBC cases paired with primary tumor and liver metastatic nodules (GSE132223), and we found that FABP1 was also located in the up-regulated DEGs dataset (Figure 1D). FABP1 mRNA transcript levels were significantly upregulated in both liver metastatic nodules and with DI compared with tumors *in situ*. Thus, FABP1 may play an important role in liver metastasis of GBC.

### High expression of FABP1 in DI region

**Figure 2:**
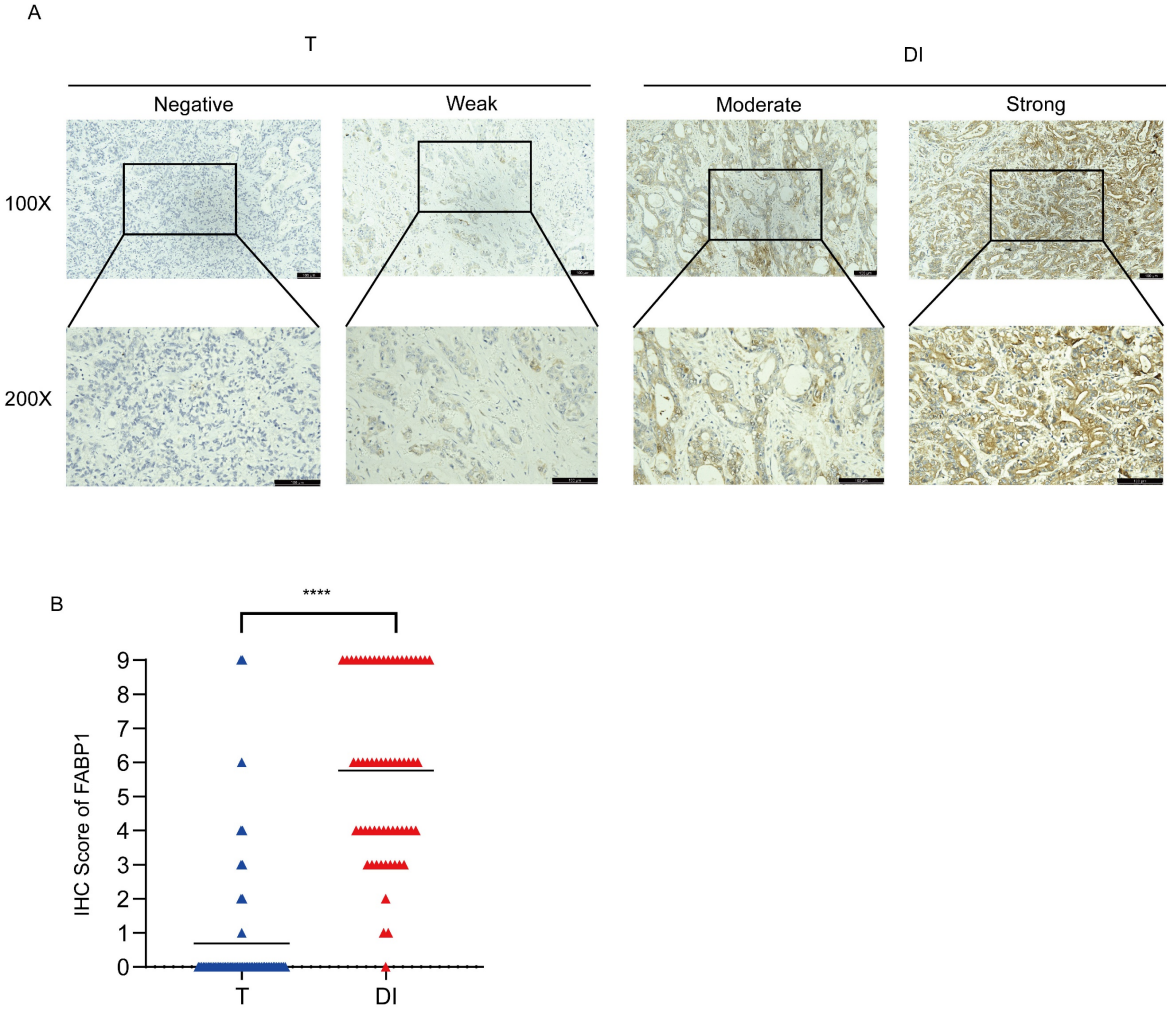
Differential expression of FABP1 in GBC tissues. **(A)** Representative images of IHC staining of FABP1 in T and DI region (100X and 200X). Bar: 100 um. Different level analyses of FABP1 expression were described in the Methods. **(B)** Comparative analysis the IHC scores of different regions. ****P < 0.0001.

To further verify the protein expression levels of FABP1 in the T and DI regions, we stained FABP1 on 62 samples with liver invasion. Representative images of IHC staining for FABP1 were shown in Figure 2A. IHC analysis showed that in T region, majority of these cases (52, 83.9%) did not express FABP1, and 3 (4.8%) and 4 (6.5%) samples expressed weak and moderate levels of FABP1, respectively. Of not, only 3 (4.8%) tumors expressed strong FABP1. Contrarily, in DI area, 35 cases (56.5%) expressed strong FABP1, whereas 23 (37.1%), 3 (4.8%) and 1 (1.6%) cases were moderate, weak and negative, respectively. Statistical analysis showed that the expression level of FABP1 in DI region was significantly higher than that in T (p < 0.0001) region (Figure 2B). These results suggest that FABP1 overexpression in the DI region may play a pathologic role in the invasion of GBC in the liver.

### Correlation of FABP1 with clinicopathological parameters and survival

We then analyzed the potential association between the expression levels of FABP1 in the T or DI region and clinicopathological characteristics of GBC patients. The mean IHC score for FABP1 was used as the cut-off value to divide patients into high and low expression groups. The mean IHC score for FABP1 in the T region was 0.69 (range 0-9), whereas in the DI region, it was 5.75 (range 0-9). The correlation analysis between FABP1 expression levels and the clinicopathological features of GBC patients demonstrated that FABP1 expression level in DI region was significantly correlated with lymph node metastasis (P=0.028), but not correlated with other clinical factors such as patient age, gender, tumor size, degree of differentiation and nerve invasion (P>0.05) (Supplementary Table S3).

**Figure 3:**
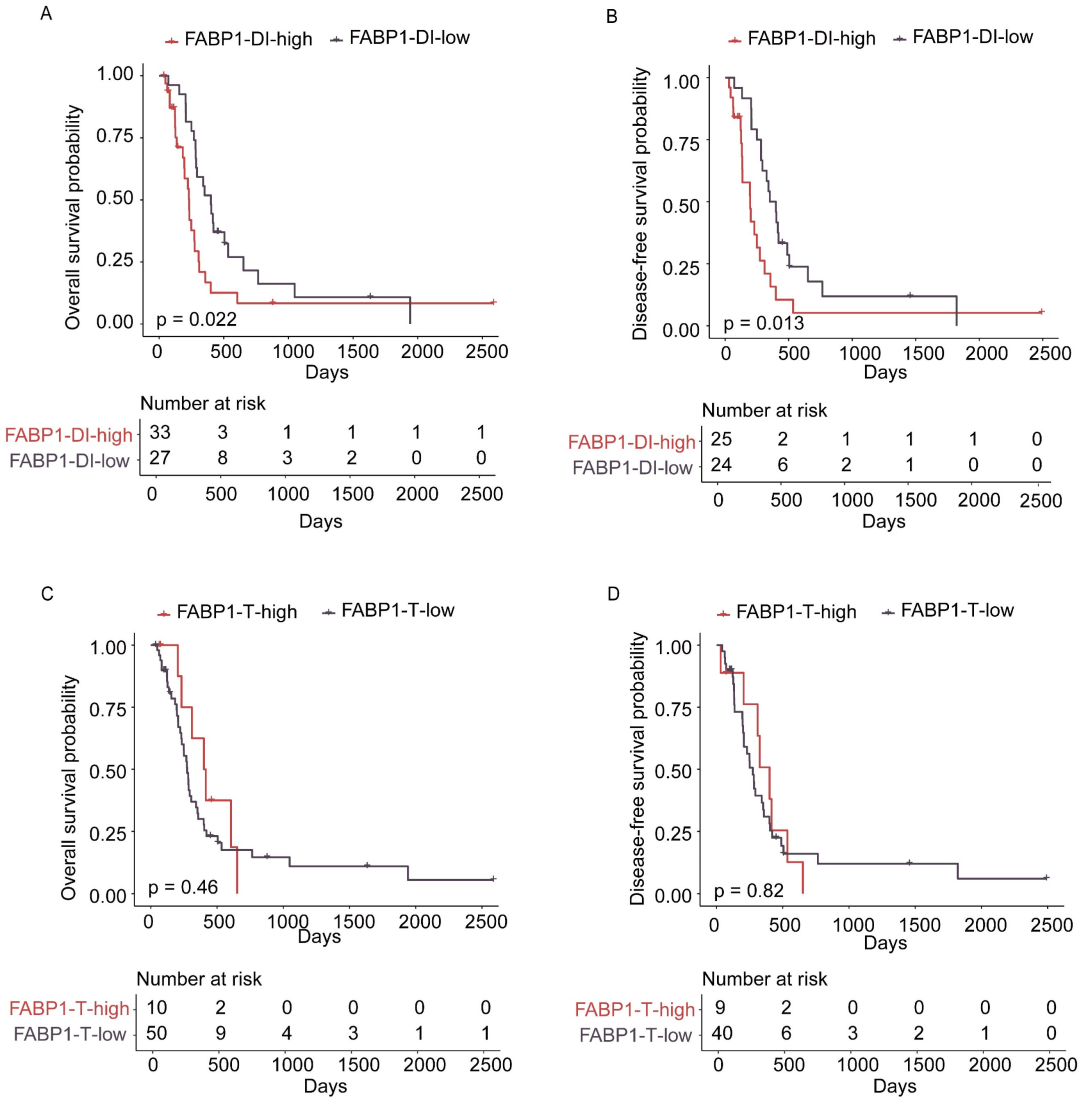
Association of survival with FABP1 expression in the T and DI regions. **(A-B)** Kaplan-Meier analysis of overall survival (OS; A) and disease-free (DFS; B) survival of patients with high and low levels of FABP1 in DI region. **(C-D)** OS (C) and DFS (D) of patients with high and low levels of FABP1 expression in T region.

However, no correlation was found between expression levels of FABP1 in the T region and any clinical features (Supplementary Table S4). Interestingly, 35 out of 62 GBC expressed high levels of FABP1 in DI region, which include 28 out of 49 adenocarcinomas, 1 out of 5 neuroendocrine carcinomas, 2 out of 4 adenosquamous carcinomas, and all of 2 mixed adenoneuroendocrine carcinomas and 2 undifferentiated carcinomas. It is evident that elevated FABP1 expression in the DI region is not limited to adenocarcinoma subtype of GBC; rather, it is also observed in other histological subtypes of GBC. However, due to the relatively low incidence of other histological subtypes of GBC, the limited sample size in our current study may not be sufficient to fully elucidate the correlation between FABP1 expression and GBC histological subtypes. We then analyzed the prognostic value of FABP1 expression in GBC using Kaplan-Meier survival curves and log-rank test analyses. The results indicated that elevated FABP1 protein expression in DI region was significantly correlated with decreased OS (P=0.022) and DFS (P=0.013) (Figure 3A-B). The median OS in the low and high FABP1 group were 400 days and 232 days, respectively. Likewise, the median DFS in the low and high FABP1 group were 376 days and 197 days, respectively. A significantly poorer prognosis was detected in patients with higher FABP1 expression in DI region. However, neither high nor low FABP1 protein expression in the T region was statistically associated with patient overall or disease-free survival (OS: p=0.46; DFS: p=0.82; Figure 3C-D). Thus, the increased expression of FABP1 in the tumor leading edge of liver invasion implies the pathological function associated with the malignant transformation of the disease.

### Correlation of TILs density in DI area with clinical factors and prognosis

**Figure 4:**
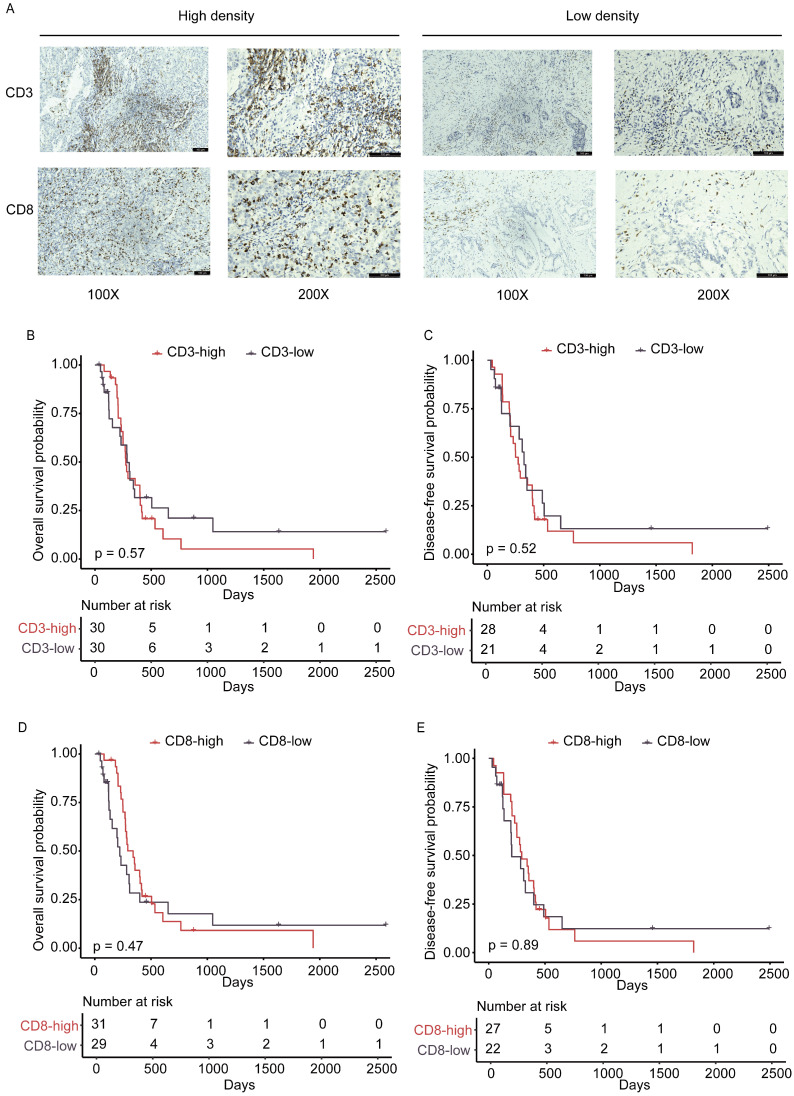
The density and prognostic significance of TILs in DI area. **(A)** Representative IHC staining images of high and low densities for CD3^+^ and CD8^+^ TILs. n=31 for each group. **(B-C)** Kaplan-Meier analysis of overall survival (OS; B) and disease-free (DFS; C) survival based on high and low levels of CD3^+^ tumor-infiltrating lymphocytes (TILs). **(D-E)** Kaplan-Meier analysis of OS (D) and DFS (E) survival based on high and low levels of CD8^+^ TILs.

While analyzing the HE-stained images, we found varied changes of lymphocytic cell infiltration in the DI field. Growing evidence has established that T lymphocytes, especially CD8^+^ cytotoxic T lymphocytes (CTL), function to protect tumor development as the main anti-tumor immune effector cells 30, 31. The prognostic value of CD3^+^ and CD8^+^ TILs in patients with GBC has been reported in a number of studies, including our previous publications 32-34, but the clinical significance and prognostic value of TILs homing to the front liver area have not been investigated. Thus, the altered staining of TILs infiltration had encouraged us to determine if their infiltration is associated with GBC hepatic invasion or FABP1 expression. The density of CD8^+^ and CD3^+^ immune cells in the DI was evaluated using IHC image analyses. Representative immunehistochemical results of different densities of immune cells in the DI region were shown in Figure 4A. The median density of two types of CD3^+^ and CD8^+^ immune cells with 710.4 cells/mm^2^ and 245.6 cells/mm^2^, respectively, was used as their cut-off values for analyzing their association with the clinicopathological characteristics (Supplementary Table S5-S6). There were no statistically significant differences in clinicopathological factors between the high and low density of CD3^+^ or CD8^+^ TILs groups. The KM curves illustrated that there was no significant correlation between any of the two TILs subtypes and postoperative recurrence or survival (DFS and OS, Figure 4B-E). Thus, the study analyses indicate that infiltration of CD3^+^ and CD8^+^ T cells in DI area does not correlate with liver invasion and cancer malignancy.

### Correlation of TILs density in DI area with FABP1 expression

**Figure 5:**
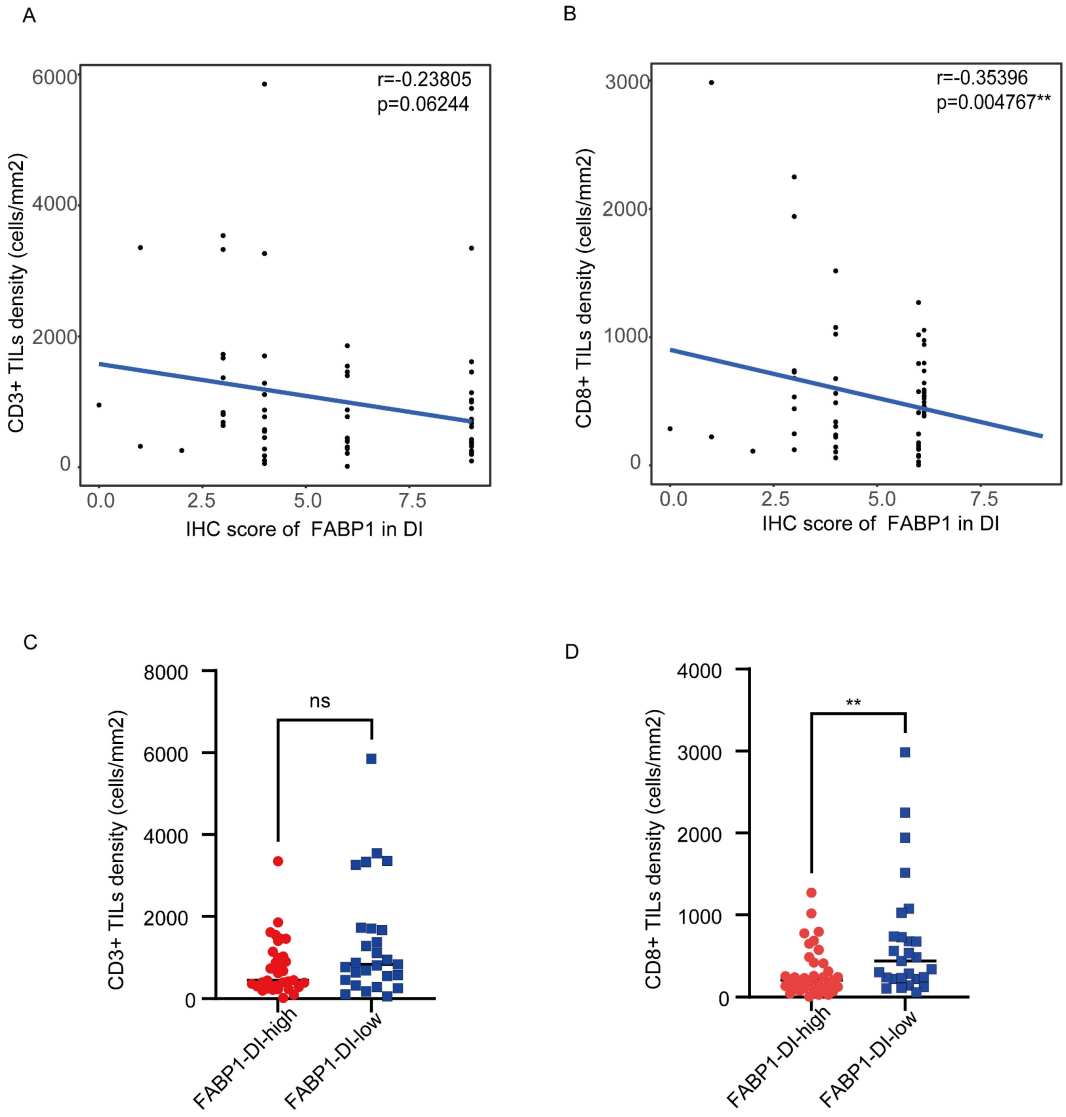
Correlation of TILs density in DI area with FABP1 expression. **(A-B)** FABP1 score correlation analysis with CD3^+^ TILs (A) and CD8^+^ TILs (B) density in DI region. **(C-D)** Differences in CD3^+^ TILs (C) and CD8^+^ TILs (D) density between patients with high and low FABP1 expression (n=62). ns: non-significant. **P < 0.01.

To investigate the potential relationship between FABP1 and CD3^+^ and CD8^+^ in the DI region, we performed correlation analysis between the density of these TILs and FABP1 IHC score. CD8^+^ TILs density was negatively correlated with the expression levels of FABP1 (r = -0.35396, P = 0.004767), while there was no significant correlation between CD3^+^ TILs density and FABP1 expression level (Figure 5A-B). Subsequently, the CD3^+^ and CD8^+^ TILs densities of patients in high FABP1 group and low FABP1 group were compared respectively (Figure 5C-D). CD8^+^ TILs density was significantly higher in patients with low FABP1 expression than those with high expression (P=0.0084). These results suggest that the increased expression of FABP1 is significantly associated with decreased infiltration of CD8^+^ cells in DI region. FABP1 may play a role in inhibiting the cytotoxicity of CD8^+^ T lymphocytes that mediate anti-tumor immunity in the tumor immune microenvironment at the invasion front of the liver.

### Risk factors for OS and DFS in GBC with hepatic invasion

**Figure 6:**
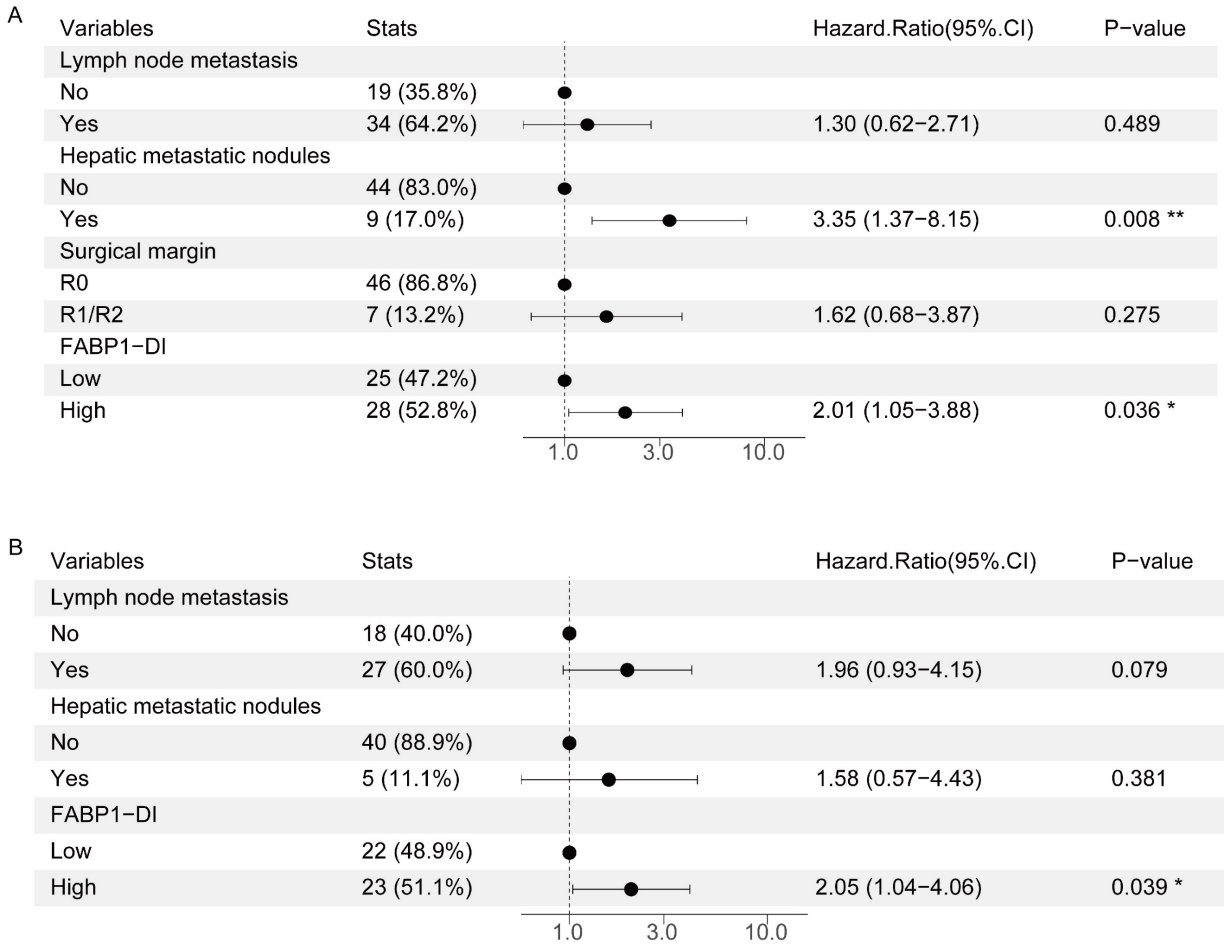
Risk factors for postoperative survival of GBC with DI. **(A-B)** Multivariate analysis of factors associated with OS(A) or DFS(B). *P < 0.05 **P < 0.01.

A previous clinic trial reported that hepatic invasion of GBC was an independent risk factor for poor disease prognosis 35. To further interrogate if other risk factor(s) contribute to the poor prognosis of GBC with DI, we measured 16 variables for univariate analysis. This analysis revealed that none of variables including age, sex, gallstone, tumor size, tumor status, lymph node metastasis, distant metastasis, AJCC stage, histologic subtype, tumor differentiation, and perineural invasion was significantly correlated with poor prognosis in GBC patients with DI (Supplementary Table S7). However, hepatic metastatic nodules (HR = 2.37, 95%CI: 1.20-4.69, P = 0.013), surgical margin (HR = 2.26, 95%CI: 1.07-4.77, P = 0.033), and FABP1 expression in DI region (HR = 1.97, 95%CI: 1.09-3.56, P = 0.024) were associated with a poor prognosis. In multivariate analysis, hepatic metastatic nodules (HR = 3.35, 95%CI: 1.37-8.15, P = 0.008) and FABP1 expression in DI region (HR = 2.01, 95%CI: 1.05-3.88, P = 0.036) were independent risk factors of OS (Figure 6A).

In the analysis of the risk factors in DFS, the univariate analyses showed that lymph node metastasis (HR = 2.09, 95%CI: 1.03-4.25, P = 0.042), hepatic metastatic nodules (HR = 2.62, 95%CI: 1.09-6.30, P = 0.032) and FABP1 expression in DI region (HR = 2.21, 95%CI: 1.17-4.18, P = 0.015) were all associated with DFS in GBC patients (Supplementary Table S8). Additionally, multivariate analysis further demonstrated that only FABP1 expression in DI region (HR = 2.05, 95%CI: 1.04-4.06, P = 0.039) was an independent prognostic indicator for DFS of GBC patients (Figure 6B). Overall, our results suggest that elevated FABP1 expression in DI region is an independent risk factor for poor prognosis of GBC with DI.

## Discussion

Liver metastasis is the most common malignancy for patients with GBC, in which direct liver invasion is one of the predominant manners in the malignant transformation. Although there are accumulating studies that focus on the pathogenesis of liver metastasis of GBC, the substantial insight into aberrant expression of molecules that serve as prognostic biomarkers in clinical practice is largely limited. Here we reported that FABP1 was highly elevated in advanced GBC with DI. Interestingly, we found that FABP1 mRNA levels were significantly upregulated in DI region compared to T region. Accordingly, IHC analysis confirmed that FABP1 expressed in DI area was higher than in T area and was significantly correlated with lymph node metastasis. Multivariate COX regression model with OS identified hepatic metastatic nodules and FABP1 expression level as independent risk factors for patient prognosis. In addition, there was a significant negative correlation between FABP1 expression level and CD8^+^ TILs density in the DI region. This is the first study to identify FABP1 expression levels in area of liver invasion, suggesting that FABP1 serves as a prognostic biomarker for the progression of GBC with liver invasion.

Multiple clinicopathological factors are known to be associated with clinical outcomes and prognosis of GBC, including TNM stage 36, extent of resection, lymph node involvement 37, distant metastasis 38, histologic differentiation 39, nerve invasion 39, 40, and involvement of resection margin. However, in GBC patients with advanced stage 3 and 4 in the absence of distant metastasis, bleeding, poor histology, liver invasion, and ≥4 regional lymph node metastases were found to be independent prognostic factors for poor prognosis 35. Interestingly, our current study with all liver invaded cases unveiled that these above clinicopathological factors failed to predict the prognosis; but only liver nodule metastasis was correlated with poor survival. Indeed, agreed with our findings, a clinical study on 42 patients with liver spread of GBC showed that liver metastasis was an independent prognostic factor for survival and recurrence in patients with GBC after hepatectomy, and these patients with hepatic metastatic nodules had a worse prognosis 9. In addition, we also identified that FABP1, a specific molecule for liver metastasis in DI region was an independent risk factor for OS and DFS; whereas TNM stage, tissue differentiation and nerve invasion were not risk factors. Therefore, elevated levels of FABP1 in DI region serve as a valuable prognostic biomarker and a potential target for therapy.

There is accumulating evidence indicating that FABP1 is up-regulated in a variety of carcinomas, such as prostate, liver and pancreas cancers, although down-regulated in other cancers 41, 42. For example, increased levels of FABP1 were reported in the early stage of gastric cancers, with a specificity of 95%, and in the advanced stage of cancer recurrence with a sensitivity of 67%, demonstrating a potential biomarker for the worse prognosis 43. Agreed with these studies, our current research showed that FABP1 expression was significantly higher in DI region than in T area. Given that FABP1 acts as an essential transport to mediate fat acid translocation and lipid metabolism in cancers, we speculate the same function most likely played by FABP1during liver invasion, where highly proliferated tumor cells disseminate and develop liver colonization. Therefore, our further studies will primarily focus on the functional and molecular mechanisms of FABP1 underlying the progression of GBC with liver invasion.

Although our initial screening samples (3 cases) and following examination using 62 GBC patients have given rise to our expected results, a larger volume with more GBC cohorts from multiple cancer centers are essential for establishing FABP1 as a prognostic biomarker in GBC patients with direct hepatic invasion. In addition, the cellular and mechanistic insight into impaired immune cell infiltration remains to be fully understood. Nevertheless, our present study has revealed the pathological impact of FABP1 on hepatic invasion of GBC.

## Supplementary Material

Supplementary tables.

## Abbreviations

GBC: Gallbladder cancer
FABP1: fatty acid binding protein 1
DI: Direct liver invasion
TILs: tumor-infiltrating lymphocytes
IHC: immunehistochemical
DFS: disease-free survival
OS: overall survival
DEGs: differentially expressed genes
TILs: Tumor-infiltrating lymphocytes
