# Supplementary material for: Fatty Acid Binding Protein 1 is an Independent Prognostic Biomarker for Gallbladder Cancer with Direct Hepatic Invasion
Source: Int J Med Sci. 2024 Mar 25;21(5):862–73. doi: 10.7150/ijms.93413 (PMC11008480; doi:10.7150/ijms.93413)
Supplement: Supplementary file 1 — Supplementary tables. [file ijmsv21p0862s1.pdf]

Supplementary Table S1. Antibodies list

Antibody name	Source	Catalog number	Dilutability
FABP1	Santa cruz	sc-271591	1:900
CD3	Proteintech	17617-1	1:300
CD8	Abcam	ab237710	1:500

Supplementary Table S2. Patients' demographics and clinicopathologic characteristics of 62 GBC with direct liver invasion

Characteristic	Value (%)
Age(y)	
Median (IQR)	67 (60-71)
Sex	
Male (%)	26 (41.94%)
Female (%)	36 (58.06%)
History of gallstone	
Yes (%)	30 (48.39%)
No (%)	32 (51.61%)
Tumor size (cm)	
Median (IQR)	4.00 (3.50-6.00)
Tumor status	
pT3(%)	50 (80.65%)
pT4(%)	12 (19.35%)
Nodal status	
N0(%)	19 (34.55%)
N1/N2(%)	36 (65.45%)
Distant metastasis	
M0(%)	36 (58.06%)
M1(%)	26 (41.94%)
AJCC 8th TNM stage	
III (%)	28 (45.16%)
IV (%)	34 (54.84%)
Histologic subtype	
Adenocarcinoma (%)	49 (79.03%)
Variants (%)	13 (20.97%)
Tumor differentiation	
I-II (%)	21 (65.62%)
III-IV (%)	11 (34.38%)
Surgical margin	
R0(%)	53 (85.48%)
R1/R2(%)	9 (14.52%)
Perineural invasion	
Yes (%)	28 (45.16%)
No (%)	34 (54.84%)
Liver nodule metastasis	
Yes (%)	13 (20.97%)
No (%)	49 (79.03%)

TNM, tumor-node-metastasis.

Supplementary Table S3. Association between FABP1 expression in DI region and clinicopathological features of patients

Characteristic	Level	Low (n=27)	High (n=35)	P-value
Age(y)	Median (IQR)	67.00 (61.50 to 72.00)	65.00 (59.50 to 70.00)	0.414
Sex	female	14 (51.9%)	22 (62.9%)	0.541
	male	13 (48.1%)	13 (37.1%)	
History of gallstone	No	18 (66.7%)	14 (40%)	0.068
	Yes	9 (33.3%)	21 (60%)	
Tumor size (cm)	Median (IQR)	4.00 (3.00 to 6.00)	5.00 (3.50 to 6.00)	0.486
Tumour status	T3	20 (74.1%)	30 (85.7%)	0.409
	T4	7 (25.9%)	5 (14.3%)	
Nodal status	N0	13 (52%)	6 (20%)	0.028*
	N1/N2	12 (48%)	24 (80%)	
Distant metastasis	M0	19 (70.4%)	17 (48.6%)	0.143
	M1	8 (29.6%)	18 (51.4%)	
AJCC 8th TNM stage	III	13 (48.1%)	15 (42.9%)	0.875
	IV	14 (51.9%)	20 (57.1%)	
Histologic subtype	Variants	6 (22.2%)	7 (20%)	1.000
	Adenocarcinoma	21 (77.8%)	28 (80%)	
Tumor differentiation	I-II	11 (73.3%)	10 (58.8%)	0.625
	III-IV	4 (26.7%)	7 (41.2%)	
Perineural invasion	No	17 (63%)	17 (48.6%)	0.383
	Yes	10 (37%)	18 (51.4%)	
Liver nodule metastasis	No	23 (85.2%)	26 (74.3%)	0.465
	Yes	4 (14.8%)	9 (25.7%)	
Surgical margin	R0	24 (88.9%)	29 (82.9%)	0.760
	R1/R2	3 (11.1%)	6 (17.1%)	

TNM, tumor-node-metastasis. *p<0.05

Supplementary Table S4. Association between FABP1 expression in T region and clinicopathological features of patients

Characteristic	Level	Low (n=52)	High (n=10)	P-value
Age (y)	Median (IQR)	66.50 (60.50 to 71.50)	68.00 (59.00 to 70.00)	0.737
Sex	female	31 (59.6%)	5 (50%)	0.830
	male	21 (40.4%)	5 (50%)	
History of gallstone	No	26 (50%)	6 (60%)	0.815
	Yes	26 (50%)	4 (40%)	
Tumor size (cm)	Median (IQR)	4.50 (3.25 to 6.00)	4.00 (3.50 to 6.00)	0.777
Tumour status	T3	44 (84.6%)	6 (60%)	0.172
	T4	8 (15.4%)	4 (40%)	
Nodal status	N0	16 (35.6%)	3 (30%)	1.000
	N1/N2	29 (64.4%)	7 (70%)	
Distant metastasis	M0	29 (55.8%)	7 (70%)	0.627
	M1	23 (44.2%)	3 (30%)	
AJCC 8th TNM stage	III	26 (50%)	2 (20%)	0.162
	IV	26 (50%)	8 (80%)	
Histologic subtype	Variants	13 (25%)	0 (0%)	0.176
	Adenocarcinoma	39 (75%)	10 (100%)	
Tumor differentiation	I-II	18 (72%)	3 (42.9%)	0.325
	III-IV	7 (28%)	4 (57.1%)	
Perineural invasion	No	29 (55.8%)	5 (50%)	1.000
	Yes	23 (44.2%)	5 (50%)	
Liver nodule metastasis	No	41 (78.8%)	8 (80%)	1.000
	Yes	11 (21.2%)	2 (20%)	
Surgical margin	R0	44 (84.6%)	9 (90%)	1.000
	R1/R2	8 (15.4%)	1 (10%)	

TNM, tumor-node-metastasis.

Supplementary Table S5. Correlation analysis between $CD3^+$ TILs density and clinical characteristics of patients

Characteristic	Level	Low density (n=31)	High density (n=31)	P-value
Age (y)	Median (IQR)	65.00 (60.50 to 71.50)	67.00 (60.50 to 70.50)	0.927
Sex	female	14 (45.2%)	22 (71%)	0.072
	male	17 (54.8%)	9 (29%)	
History of gallstone	No	13 (41.9%)	19 (61.3%)	0.204
	Yes	18 (58.1%)	12 (38.7%)	
Tumor size (cm)	Median (IQR)	5.75 (3.50 to 8.00)	4.00 (3.25 to 5.00)	0.062
Tumor status	T3	24 (77.4%)	26 (83.9%)	0.748
	T4	7 (22.6%)	5 (16.1%)	
Nodal status	N0	10 (34.5%)	9 (34.6%)	1.000
	N1/N2	19 (65.5%)	17 (65.4%)	
Distant metastasis	M0	17 (54.8%)	19 (61.3%)	0.797
	M1	14 (45.2%)	12 (38.7%)	
AJCC 8th TNM stage	III	12 (38.7%)	16 (51.6%)	0.444
	IV	19 (61.3%)	15 (48.4%)	
Histologic subtype	Variants	8 (25.8%)	5 (16.1%)	0.533
	Adenocarcinoma	23 (74.2%)	26 (83.9%)	
Tumor differentiation	I-II	11 (68.8%)	10 (62.5%)	1.000
	III-IV	5 (31.2%)	6 (37.5%)	
Perineural invasion	No	18 (58.1%)	16 (51.6%)	0.799
	Ye	13 (41.9%)	15 (48.4%)	
Liver nodule metastasis	No	25 (80.6%)	24 (77.4%)	1.000
	Yes	6 (19.4%)	7 (22.6%)	
Surgical margin	R0	24 (77.4%)	29 (93.5%)	0.149
	R1/R2	7 (22.6%)	2 (6.5%)	

TNM, tumor-node-metastasis.

Supplementary Table S6. Correlation analysis between $CD8^+$ TILs density and clinical characteristics of patients

Characteristic	Level	Low density (n=30)	High density (n=32)	P-value
Age (y)	Median (IQR)	68.00 (59.00 to 70.00)	65.50 (60.50 to 71.50)	1.000
Sex	female	15 (50%)	21 (65.6%)	0.323
	male	15 (50%)	11 (34.4%)	
History of gallstone	No	13 (43.3%)	19 (59.4%)	0.313
	Yes	17 (56.7%)	13 (40.6%)	
Tumor size (cm)	Median (IQR)	5.00 (4.00 to 8.00)	4.00 (2.75 to 5.50)	0.101
Tumor status	T3	25 (83.3%)	25 (78.1%)	0.844
	T4	5 (16.7%)	7 (21.9%)	
Nodal status	N0	9 (32.1%)	10 (37%)	0.922
	N1/N2	19 (67.9%)	17 (63%)	
Distant metastasis	M0	17 (56.7%)	19 (59.4%)	1.000
	M1	13 (43.3%)	13 (40.6%)	
AJCC 8th TNM stage	III	12 (40%)	16 (50%)	0.592
	IV	18 (60%)	16 (50%)	
Histologic subtype	Variants	8 (26.7%)	5 (15.6%)	0.450
	Adenocarcinoma	22 (73.3%)	27 (84.4%)	
Tumor differentiation	I-II	10 (66.7%)	11 (64.7%)	1.000
	III-IV	5 (33.3%)	6 (35.3%)	
Perineural invasion	No	18 (60%)	16 (50%)	0.592
	Yes	12 (40%)	16 (50%)	
Liver nodule metastasis	No	24 (80%)	25 (78.1%)	1.000
	Yes	6 (20%)	7 (21.9%)	
Surgical margin	R0	24 (80%)	29 (90.6%)	0.409
	R1/R2	6 (20%)	3 (9.4%)	

TNM, tumor-node-metastasis.

Supplementary Table S7. Univariate analysis of factors affecting overall survival

Variables	Hazard Ratio (95% CI)	P-value
Age (>60 vs. ≤60 years)	0.97 (0.50-1.89)	0.930
Sex (male vs. female)	0.68 (0.37-1.24)	0.208
History of gallstone (yes vs. no)	0.77 (0.43-1.40)	0.393
Tumor size (≥ 4 vs. <4 cm)	1.06 (0.59-1.91)	0.845
Tumor status (T4 vs. T3)	0.82 (0.41-1.67)	0.587
Distant metastasis (M1 vs. M0)	1.39 (0.77-2.51)	0.268
AJCC 8th TNM stage (IV vs. III)	1.21 (0.67-2.18)	0.525
Histologic subtype (Adenocarcinoma vs. others)	0.91 (0.44-1.86)	0.792
Tumor differentiation (III-IV vs. I-II)	1.03 (0.48-2.24)	0.933
Perineural invasion (yes vs. no)	0.85 (0.47-1.54)	0.596
Lymph node metastasis (yes vs. no)	1.73 (0.89-3.38)	0.109
Hepatic node metastasis (yes vs. no)	2.37 (1.20-4.69)	0.013*
Surgical margin (R1/R2 vs. R0)	2.26 (1.07-4.77)	0.033*
FABP1-DI (high vs. low)	1.97 (1.09-3.56)	0.024*

TNM, tumor to node to metastasis; FABP1-DI, FABP1 expression levels in areas of direct liver invasion. *p < 0.05

Supplementary Table S8. Univariate analysis of factors affecting disease-free survival

Variables	Hazard Ratio (95% CI)	P-value
Age (>60 vs. ≤60 years)	0.58 (0.29-1.18)	0.131
Sex (male vs. female)	0.80 (0.42-1.53)	0.503
History of gallstone (yes vs. no)	0.68 (0.36-1.29)	0.238
Tumor size (≥ 4 vs. <4cm)	0.85 (0.44-1.63)	0.621
Tumor status (T4 vs. T3)	0.88 (0.42-1.86)	0.741
Distant metastasis (M1 vs. M0)	1.25 (0.64-2.45)	0.514
AJCC 8th TNM stage (IV vs. III)	1.14 (0.61-2.12)	0.691
Histologic subtype (Adenocarcinoma vs. others)	0.93 (0.43-2.04)	0.863
Tumor differentiation (III-IV vs. I-II)	0.97 (0.39-2.41)	0.940
Perineural invasion (yes vs. no)	1.01 (0.53-1.92)	0.984
Lymph node metastasis (yes vs. no)	2.09 (1.03-4.25)	0.042*
Hepatic node metastasis (yes vs. no)	2.62 (1.09-6.30)	0.032*
FABP1-DI (high vs. low)	2.21 (1.17-4.18)	0.015*

TNM, tumor to node to metastasis; FABP1-DI, FABP1 expression levels in areas of direct liver invasion. *$p<0.05$
